# Insane Pauper Importation

**Published:** 1887-07

**Authors:** 


### INSANE PAUPER IMPORTATION

Too much praise cannot be accorded to the Collector of the Port at Boston for his stand concerning the importation of insane paupers. The steamship Cephalonia, of the Cunard line, arrived at Boston bringing among its passengers a woman bearing every evidence of insanity, though not of a violent type. The Superintendent of the State Board of Lunacy and Charity refused to allow her to land, as by so doing she would become a burden on the state. The officers secretly disposed of the woman, and the company was notified that unless she was returned clearance papers would not be granted. The matter was brought to the notice of the Collector. The hearing was private and did not occupy much time, as it was evident that the company had violated the statutes. In making his decision the Collector said: "I shall impose a fine of $1,000 on the Cunard Steamship Company for landing at this port an insane person named Johannah Casey. Whenever I receive a check for that amount I will furnish the necessary papers for the clearance of the steamer Cephalonia, and not until then. Before that check is used the company will be given an opportunity of presenting the case to United States Secretary of the Treasury Fairchild, and I shall, of course, be governed by his decision."

There can be no doubt of the justice of this decision. The authorities should no longer permit this country to be made an asylum for the paupers, the insane, and the general riff-raff from every nation on the face of the earth. It is to be hoped that the Secretary of the Treasury will sustain the decision of the Collector. There is abundant evidence that many of the countries across the water actually pay the passage rate to this country for the vagrant and pauper population rather than support them at home, and up to this time there has been no one to say them nay. The decision in the above case is one that should be followed up in many similar cases which occur at the different ports every day.

